# Diagnosis and management of inhalation injury: an updated review

**DOI:** 10.1186/s13054-015-1077-4

**Published:** 2015-10-28

**Authors:** Patrick F. Walker, Michelle F. Buehner, Leslie A. Wood, Nathan L. Boyer, Ian R. Driscoll, Jonathan B. Lundy, Leopoldo C. Cancio, Kevin K. Chung

**Affiliations:** Department of Surgery, Walter Reed National Military Medical Center, 8901 Wisconsin Ave, Bethesda, MD 20889 USA; Department of General Surgery, San Antonio Military Medical Center, 3551 Roger Brooke Dr., Fort Sam Houston, TX 78234 USA; Department of Medicine, San Antonio Military Medical Center, 3551 Roger Brooke Dr., Fort Sam Houston, TX 78234 USA; United States Army Institute of Surgical Research, Fort Sam Houston, TX 78234 USA; Department of Surgery, Uniformed Services University of the Health Sciences, Building A, 4301 Jones Bridge Rd, Bethesda, MD 20814 USA

## Abstract

In this article we review recent advances made in the pathophysiology, diagnosis, and treatment of inhalation injury. Historically, the diagnosis of inhalation injury has relied on nonspecific clinical exam findings and bronchoscopic evidence. The development of a grading system and the use of modalities such as chest computed tomography may allow for a more nuanced evaluation of inhalation injury and enhanced ability to prognosticate. Supportive respiratory care remains essential in managing inhalation injury. Adjuncts still lacking definitive evidence of efficacy include bronchodilators, mucolytic agents, inhaled anticoagulants, nonconventional ventilator modes, prone positioning, and extracorporeal membrane oxygenation. Recent research focusing on molecular mechanisms involved in inhalation injury has increased the number of potential therapies.

## Introduction

Despite important advances in the care of patients with inhalation injury, which continues to be largely supportive, morbidity and mortality remain high [1]. Inhalation injury can feature supraglottic thermal injury, chemical irritation of the respiratory tract, systemic toxicity due to agents such as carbon monoxide (CO) and cyanide, or a combination of these insults. The resultant inflammatory response may cause higher fluid resuscitation volumes, progressive pulmonary dysfunction, prolonged ventilator days, increased risk of pneumonia, and acute respiratory distress syndrome (ARDS) [2, 3].

In this review we describe the recent advances made in our understanding of the pathophysiology of inhalation injury, diagnostic criteria and injury severity, complications, current treatment options, and future avenues of research.

## Pathophysiology

Inhalation injury complicates burns in approximately 10 to 20 % of patients and significantly increases morbidity and mortality [2–5]. Other factors associated with a significant effect on mortality include burn size and age [6, 7] and the incidence of inhalation injury is correlated with an increase in both these factors [6, 7]. Inhalation injury has also been found to be an independent predictor of mortality in burn patients [8] and worsens survival even among patients with similar age and burn size [8]. Thermal airway injury is generally limited to supraglottic structures, whereas injury to the lower airway is chemical in nature. In the setting of steam, however, the injury is pervasive, causing damage to both upper airways and direct thermal injury to the lungs [9]. The degree of inhalation injury is variable and is dependent on several factors: the gas components inhaled, the presence of particulate matter (soot), the magnitude of the exposure, and individual host factors such as underlying lung disease and inability to flee the incident.

Historically, it was speculated that the combustion of certain materials, such as noncommercial polyurethane foam, resulted in the formation of a neurotoxin [10]; however, newer material testing methods revealed most smoke toxicity can be explained by a small number of toxic gases exerting their effects through asphyxiation, systemic toxicity, or direct effects on respiratory tissue [11]. Many products of combustion, such as carbon dioxide, function as simple asphyxiants by displacing oxygen at the alveolar level. This is further exacerbated by the hypoxic fire environment. CO functions systemically as an asphyxiant by (1) competitively displacing oxygen from hemoglobin and (2) binding to cytochrome oxidase at the mitochondrial level. By contrast, hydrogen cyanide binds only to cytochrome oxidase [1].

Other combustion products, such as halogen acids, formaldehyde, and unsaturated aldehydes (for example, acrolein), function as respiratory irritants. The chemical irritation causes denuding of the respiratory mucosa, leading to sloughing within the airways, and induces the host inflammatory response. Furthermore, the chemical injury stimulates vasomotor and sensory nerve endings to produce neuropeptides. Preclinical studies have shown these neuropeptides can induce an inflammatory response [12]. Substance P and calcitonin gene-related peptide are two suggested neuropeptides inducing tissue injury after inhalation injury [12, 13]. Lange *et al*. [13] found antagonists to calcitonin gene-related peptide and substance P attenuated the fluid shifts/inflammation in an ovine model subjected to smoke and inhalation injury. These neuropeptides then induce bronchoconstriction and nitric oxide synthase (NOS) to generate reactive oxygen species (ROS) [14]. As described by Kraneveld and Nijkamp [14], these neuropeptides can function as tachykinins, inducing a robust inflammatory response with the downstream effects of bronchoconstriction, increased vascular permeability, and vasodilation. Furthermore, tachykinins like substance P and neurokinin A can modulate immune cells and stimulate neutrophil and eosinophil chemotaxis [14].

Overall, these factors potentiate local cellular damage and the loss of hypoxic pulmonary vasoconstriction. The loss of hypoxic pulmonary vasoconstriction causes bronchial blood flow to increase by a factor of 10 within 20 min of inhalation injury. ROS may also induce mitochondrial dysfunction and cellular apoptosis [15]. Tissue factor expressed by damaged respiratory epithelial cells and alveolar macrophages initiates the extrinsic coagulation cascade, disrupting pro- and anti-coagulant alveolar homeostasis. We found, for example, that smoke inhalation injury contributes to a hypercoagulable state in the lung by inducing plasminogen activator inhibitor 1 and stabilizing its mRNA [16].

In addition, the increased bronchial blood flow delivers activated polymorphonuclear leukocytes and cytokines to the lung, potentiating the host inflammatory response. The loss of an intact bronchial epithelium and the effects of ROS result in a loss of plasma proteins and fluid from the intravascular space into the alveoli and bronchioles [17]. The transvascular shift of protein causes exudate and cast formation within the airways, leading to alveolar collapse or complete occlusion of the airways [17]. Experimental measures to decrease bronchial blood flow show attenuation of airway obstruction, pulmonary edema, and improved oxygenation [18]. These processes - loss of hypoxic vasoconstriction, increased blood flow to injured lung segments, decreased ventilation of collapsed segments - contribute to ventilation-perfusion mismatch as a primary mechanism of hypoxemia following the smoke inhalation injury [19]. Atelectasis, dysfunction of the immune system, and mechanical ventilation, in turn, predispose to pneumonia as a common complication of inhalation injury.

Many studies are now being completed to better understand the role pro- and anti-inflammatory mediators, or other immune modulators, play in patient outcomes. Albright *et al*. [1] demonstrated a graded increase in inflammatory cytokines from bronchoalveolar lavage fluid (IL-4, IL-6, IL-9, IL-15, interferon-gamma, granulocyte macrophage colony-stimulating factor, monocyte chemoattractant protein-1) that correlates with the severity of inhalation injury noted on bronchoscopic evaluation (grades 3 or 4 versus 1 or 2). They also found a significant shift from a macrophage-predominant population of cells in lavage fluid to one dominated by neutrophils [1]. This is thought to contribute to the later immune dysfunction, bacterial overgrowth, and pneumonia [1]. The source of the cytokines identified in inhalation is thought to be secondary to complement activation by heat denatured proteins [20]. The stimulation of the complement cascade releases histamine, resulting in xanthine oxidase upregulation and ROS formation [20, 21].

Davis *et al*. [22] showed several plasma immune mediators were associated with increased inhalation injury severity, even after adjusting for age and percentage of total body surface area burned. IL-1 receptor antagonist (IL-1RA), an anti-inflammatory immune mediator, had the strongest correlation with injury severity and outcome measures, including mortality [22]. The authors also found a much lower IL-1β to IL-1RA ratio in patients with inhalation injury who died. Given that IL-1β is an essential component of the host defense, they hypothesized that insufficient IL-1β or excessive IL-1RA results in systemic immune dysfunction.

The formation of ROS, such as superoxide anions (O_2_^−^), hydrogen peroxide (H_2_O_2_), and hydroxyl radicals (OH^−^; the most unstable and reactive), appears to play a major role in numerous injury models [23]. Under normal circumstances the body has compensatory antioxidant mechanisms to mitigate the effects of ROS. Following reperfusion or injury, however, there is a large burst of ROS, which overwhelm the body’s protective measures. As a result, the ROS can lead to cell injury through neutrophil attraction and cytokine production. Indeed, evidence of oxidative stress is found in plasma and lung tissue following smoke inhalation injury [24]. IL-8, a potent chemokine, has been suggested to play a vital role in the initiation and progression of lung inflammation after smoke inhalation [25]. NOS-dependent formation of ROS has been studied in inhalation injury. Activated neutrophils produce large quantities of superoxide that combine with nitric oxide to produce peroxynitrite, which can damage DNA [20]. Peroxynitrite and the resultant DNA damage stimulates poly-(ADP ribose) polymerase-1 (PARP-1), a nuclear repair enzyme and coactivator of NF-ĸB, which can deplete ATP and produce cell damage [26, 27]. Combined, these factors contribute to an increase in IL-8 [27], induce NOS, neutrophil chemotaxis, and increase ROS production [20]. For this reason, heparin/acetylcysteine combinations are being utilized as ROS scavengers in inhalation injury [20].

## Diagnosis

Classically, the diagnosis of inhalation injury was subjective and made on the basis of clinical findings. When evaluating a patient with suspected inhalation injury, a clinician first reviews the history and reported mechanism to determine the likelihood of an inhalation injury. Pertinent information includes exposure to flame, smoke, or chemicals (industrial and household), duration of exposure, exposure in an enclosed space, and loss of consciousness or disability. Pertinent physical exam findings include facial burns, singed facial or nasal hair, soot or carbonaceous material on the face or in the sputum, and signs of airway obstruction including stridor, edema, or mucosal damage [3]. Older patients, and those with more extensive burns, are at increased risk of inhalation injury because of prolonged exposure to the fire environment [8].

There are several modalities for confirming inhalation injury to include fiberoptic bronchoscopy (FOB), chest computed tomography (CT), carboxyhemoglobin measurement, radionuclide imaging with ^133^Xenon, and pulmonary function testing. Many of these modalities lack sensitivity, are invasive, or are subject to significant variability between institutions. In studies by Shirani *et al*. [8], the following gradation of morbidity and mortality risk was seen in order of increasing risk: (1) patients without inhalation injury; (2) patients with inhalation injury by ^133^Xenon scan only, but not by FOB; and (3) patients with inhalation injury by FOB. Also, presence of inhalation injury on FOB predicted risk of acute lung injury and the need for increased fluid resuscitation volumes. More recent studies have found a significant correlation between the severity of inhalation injury on FOB and mortality [28].

**Fig. 1:**
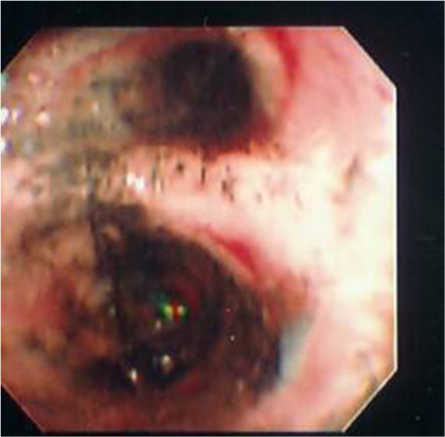
Fiberoptic bronchoscopy of patient on post-burn day 0

**Fig. 2:**
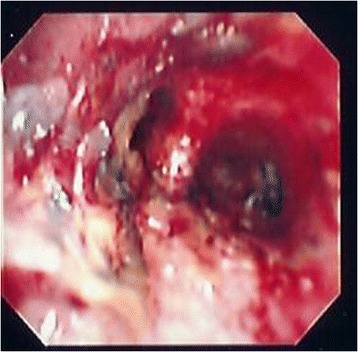
Fiberoptic bronchoscopy of patient on post-burn day 4

**Fig. 3:**
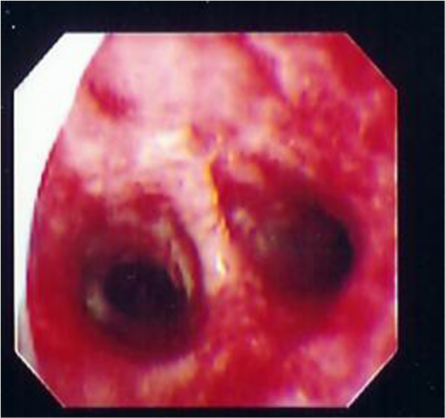
Fiberoptic bronchoscopy of patient on post-burn day 10

There are several difficulties in diagnosing the presence and severity of inhalation injuries. Although several laboratories have developed dose–response models of inhalation injury in large animals [29], the characteristics of the material inhaled are important in determining the degree of respiratory failure. In addition, differences in the individual host inflammatory response may lead to a heterogeneous clinical presentation [30]. FOB is unable to assess distal airways and respiratory bronchioles; therefore, damage to this portion of the lung has been proposed as an explanation for the discordance between bronchoscopic severity of injury and mortality. Despite these limitations, FOB continues to be the standard technique used to assess the presence and severity of inhalation injury. Its relative ease and availability allows the initial diagnosis to be made (Fig. 1), and allows the inhalation injury to be followed serially (Figs. 2 and 3).

Given the lack of a widely standardized and validated method for scoring inhalation injury severity, Woodson [30] has proposed a large multicenter study to create such a scoring system to allow for more reliable prognostic estimations. To date, no such study has been done, though one is currently underway based on clinical, radiographic, bronchoscopic, and biochemical parameters (ClinicalTrials.gov identifier NCT01194024).

Multiple studies have demonstrated that inhalation injury is a graded phenomenon with severity correlating with outcome. The Abbreviated Injury Score grading scale for inhalation injury on bronchoscopy has been shown to correlate with an increase in mortality as well impaired gas exchange [1, 28, 31]. This scale is shown in Table 1. Endorf and Gamelli [3] found that patients with more severe inhalation injury on initial bronchoscopy (grades 2, 3, 4) had worse survival rates than patients with lower scores (grades 0 or 1) (*P* = 0.03). They also noted the highest-grade inhalation injuries were not necessarily associated with an increased fluid requirement, contrary to prior data. Lastly, they found patients with an arterial partial pressure of oxygen (P_a_O_2_)/fraction of inspired oxygen (F_i_O_2_) ratio <350 upon presentation had a statistically significant increase in fluid resuscitation needs compared with patients with a ratio >350 (*P* = 0.03) [3]. Ryan *et al*. [32] have stated that, at this time, the most reliable indicator of the impact of inhalation injury is the P_a_O_2_/F_i_O_2_ ratio after the resuscitation has started. This is based on a retrospective review by Hassan *et al.* [28] of 105 patients admitted with inhalation injury. They assessed respiratory function by using the P_a_O_2_/F_i_O_2_ ratio from 0 to 192 h after injury. Their study showed a significant difference (*P* < 0.01) in P_a_O_2_/F_i_O_2_ ratios between patients who died (mean P_a_O_2_/F_i_O_2_ ratio 20.17) and those who survived (mean P_a_O_2_/F_i_O_2_ ratio 32.24). Ultimately, they propose to use P_a_O_2_/F_i_O_2_ ratio as a predictor of survival once the initial burn resuscitation has been completed and a full response to injury is able to be mounted [28]. Similarly, Cancio *et al.* [33] found that the mean alveolar-arterial oxygen gradient during the first 2 days was an independent predictor of mortality in mechanically ventilated burn patients. It is important to note that P_a_O_2_/F_i_O_2_ can be arbitrarily high or low depending on the choice of ventilator mode. In addition, P_a_O_2_/F_i_O_2_ may be affected by the volume of resuscitation. Therefore, we do not use P_a_O_2_/F_i_O_2_ as a basis for diagnosis of inhalation injury, but use it to trend the patient’s oxygenation and potential need for nonconventional ventilation.

**Table 1:** Abbreviated Injury Score grading scale for inhalation injury on bronchoscopy [1]

Grade	Class	Description
0	No injury	Absence of carbonaceous deposits, erythema, edema, bronchorrhea, or obstruction
1	Mild injury	Minor or patchy areas of erythema, carbonaceous deposits, bronchorrhea, or bronchial obstruction
2	Moderate injury	Moderate degree of erythema, carbonaceous deposits, bronchorrhea, or bronchial obstruction
3	Severe injury	Severe inflammation with friability, copious carbonaceous deposits, bronchorrhea, or obstruction
4	Massive injury	Evidence of mucosal sloughing, necrosis, endoluminal obstruction

**Fig. 4:**
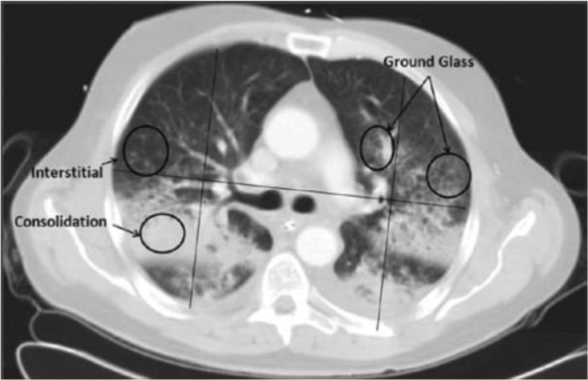
Example of radiologist’s score findings in chest computed tomography scan slice [34]

Other means of evaluating the severity of inhalation injury include chest CT. First, a scoring system for severity of CT scan findings has been developed [29]. Our group studied 25 patients with inhalation injury and 19 patients without inhalation injury who received a chest CT within 24 h of admission [34]. The severity of radiographic findings was calculated by looking at 1-cm axial slices from the chest CT and these were scored by adding the highest radiologist’s score (RADS) for each quadrant. The RADS scoring system is shown in Table 2, and the various RADS findings are shown in Fig. 4. Our group assessed a composite endpoint of pneumonia, acute lung injury/ARDS, and death. We found that the detection of inhalation injury on bronchoscopy was associated with an 8.3-fold increase in the composite endpoint. A high RADS score (>8 per slice) in addition to a positive bronchoscopy was associated with a 12.7-fold increase, thus showing the potential for chest CT to complement bronchoscopy in detecting clinically significant inhalation injury [34].

**Table 2:** Radiologist’s scoring table (RADS score) for inhalation injury [34]

Finding	Score
Normal	0
Increased interstitial markings	1
Ground glass opacification	2
Consolidation	3

Second, Yamamura *et al.* [35] used CT imaging to measure bronchial wall thickness 2 cm distal to the tracheal bifurcation in patients who had sustained an inhalation injury. The authors noted a statistically significant correlation between bronchial wall thickness and the development of pneumonia, total number of ventilator days, and ICU length of stay. They also found that a bronchial wall thickness value of >3.0 mm predicted the development of pneumonia with a sensitivity of 79 % and specificity of 96 %. Interestingly, this study was not able to replicate the association between Abbreviated Injury Score bronchoscopic scoring and clinical outcomes as described above [35].

A third approach to using the CT scan is virtual bronchoscopy. A three-dimensional reconstructed image is presented in such a way that the viewer navigates through the lung as if using a bronchoscope. We found that virtual bronchoscopy agrees best with FOB in the detection of airway narrowing, and less so in the detection of blistering or necrosis [36].

Problems with using chest CT as part of a diagnostic algorithm for inhalation injury include determining the optimal timing of the test and how to interpret abnormal radiographic findings in the setting of a negative bronchoscopy. Putman *et al.* [37] found that chest radiography on admission was rarely helpful in determining the presence or severity of inhalation injury, but its use is helpful as a baseline for determining future changes.

## Respiratory support

Given the limited availability of targeted therapies for inhalation injury, one of the fundamental tenets is supportive respiratory care. This includes aggressive pulmonary toilet and mechanical ventilation when indicated. It should be noted that approximately 20 to 33 % of patients hospitalized with inhalation injury experience some degree of upper airway obstruction due to pharyngeal edema that can progress rapidly [38]. As thermal injury increases airway edema and can lead to airway obstruction, early intubation is favored [4]. This is of particular concern in patients who receive large amounts of intravenous fluids during resuscitation. Generally speaking, the most experienced clinician in airway management should perform endotracheal intubation with the largest available, age-appropriate endotracheal tube for patients with suspected or impending upper airway obstruction in the setting of inhalation injury. One study suggests prophylactic intubation can decrease mortality related to pulmonary-related death in patients with inhalation injury [39].

Maintaining bronchial hygiene is paramount in patients who have suffered inhalation injury. Early ambulation, chest physiotherapy, airway suctioning, and therapeutic bronchoscopy are adjunctive tools [38]. Reper *et al.* [40] demonstrated that intrapulmonary percussive ventilation administered through a face mask to spontaneously breathing patients with smoke inhalation injury, hypoxia, and persistent atelectasis can result in a significant improvement in P_a_O_2_/F_i_O_2_ ratio.

A low threshold should be maintained for intubation and mechanical ventilation in inhalation injury due to the progressive nature of the airway edema. Interestingly, a study by Mackie *et al.* [41] showed an increased use of mechanical ventilation in patients at a Dutch burn center from 1997 to 2006 (76 %) compared with 1987 to 1996 (38 %) despite a decrease in the incidence of inhalation injury (34 % versus 27 %). The authors hypothesized that this was related to the institution of Advanced Trauma Life Support principles in the mid-1990s in the Netherlands. Mackie [42] also suggested mechanical ventilation may be a significant contributor to mortality in burn patients independent of inhalation injury. He proposed increased intrathoracic pressure from positive pressure ventilation led to decreased venous return, followed by decreased cardiac and urine output. The typical clinical reaction is to increase intravenous fluid administration, resulting in higher volumes of infused fluid, a known risk factor for adverse outcomes in burn patients [42].

Multiple challenges, to include concern for ventilator-induced lung injury in patients with inhalation injury, have led to the use of unconventional ventilator modes [43].

Conventional mechanical ventilation is limited in the patient with inhalation injury. In a patient with fibrin casts, extensive chest wall thermal injuries, or high volumes of resuscitative fluid maintaining the recommended tidal volumes of less than 7 ml/kg body weight and plateau pressures of less than 30 cm water [44], can prove difficult with conventional techniques. Therefore, in order to apply lung-protective ventilation in patients with inhalation injury, nonconventional ventilator modes are employed.

**Fig. 5:**
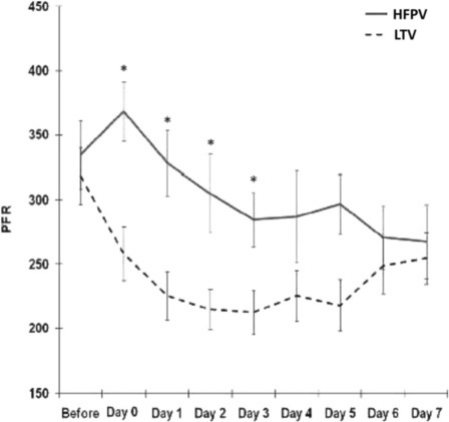
Comparison of the P_a_O_2_/F_i_O_2_ ratio over time between high frequency percussive ventilation (HFPV) and low-tidal volume ventilation (LTV) (asterisks denote *P* < 0.05) [49]

High-frequency percussive ventilation (HFPV) was first described in patients with inhalation injury as a means of assisting with clearance of sloughed respiratory mucosa and plugs, as well as decreasing iatrogenic barotrauma and the incidence of pulmonary infection [45]. Further studies have demonstrated benefits from using HFPV prophylactically (that is, not as a salvage mode) in both adult [46, 47] and pediatric populations [48]. Our group performed a randomized controlled trial to compare HFPV versus conventional low tidal volume (LTV) ventilation [49]. While we detected no difference in ventilator-free days between patients randomized to HFPV compared with LTV ventilation, there was a statistically significant increase in the P_a_O_2_/F_i_O_2_ ratio for the HFPV cohort on days 0 to 3 (Fig. 5). We also found that less patients in the HFPV cohort required conversion to a rescue mode of ventilation compared with the LTV ventilation cohort [49]. Also, HFPV was associated with a decrease in the incidence of pneumonia from 45 to 26 % (*P* < 0.005) and resulted in an improvement in survival [46]. Although HFPV cannot reverse the effects of inhalation injury, it can improve the clearance of secretions, provide positive pressure throughout the ventilator cycle, allow for lower airway pressures, and increase functional reserve capacity [46].

Interestingly, a recent retrospective study by Sousse *et al.* [50] compared high tidal volume (HTV) ventilation (15 ± 3 ml/kg, n = 190) with LTV ventilation (9 ± 3 ml/kg, n = 501) in pediatric patients suffering from inhalation injury. Patients on HTV had fewer days on the ventilator (*P* < 0.005), increased maximum peak inspiratory pressure (*P* < 0.02), and plateau pressures (*P* < 0.02) compared with those on LTV ventilation. Furthermore, the incidence of atelectasis and ARDs was significantly lower in the cohort receiving HTV ventilation (*P* < 0.0001 and *P* < 0.02, respectively). However, the HTV ventilation group were not without complications and had a significantly higher rate of pneumothorax compared with the LTV ventilation group (*P* < 0.03). For pediatric patients suffering from inhalation injury, HTV ventilation may be better than traditional LTV ventilation [50]. The mechanisms for this observation are unclear, but this study, much like the previous study, suggests that we must be cautious when extrapolating LTV ventilation to all patient populations, especially in those with different pathophysiologies. A randomized controlled trial may be necessary to tease out the true impact of these divergent strategies. Our group also looked at airway pressure release ventilation (APRV) in a prospective animal model study. We found that P_a_O_2_/F_i_O_2_ ratios were initially lower in pigs with inhalation injury on APRV compared with conventional mechanical ventilation, although this equilibrated at 48 h. Higher mean airway pressures were necessary to maintain oxygenation in APRV, and, in the end, no survival difference was seen between APRV and conventional mechanical ventilation [51].

Other nonventilator adjuncts to consider for inhalation injury include prone positioning and extracorporeal membrane oxygenation (ECMO). Our group showed that prone positioning led to a statistically significant increase in P_a_O_2_/F_i_O_2_ ratio in patients with inhalation injury and refractory ARDS whose initial ratio was an average of 87 ± 38 [52].

A systematic review and meta-analysis on the use of ECMO in inhalation injury was limited by number of studies and total patients available. There was a tendency towards increased survival in burn patients with acute hypoxemic respiratory failure treated with ECMO. ECMO use of <200 h was correlated with higher survival compared with time >200 h. There was no improvement in survival if ECMO was initiated once the P_a_O_2_/F_i_O_2_ ratio was <60 [53].

## Targeted therapies

### Bronchodilators

Bronchodilators have been used in inhalation injury to decrease airflow resistance and improve dynamic compliance. β_2_-adrenergic agonists such as albuterol and salbutamol have been studied in both sheep and humans. Ovine studies of smoke inhalation injury have shown that both nebulized epinephrine and albuterol decrease airway pressure by smooth muscle relaxation and increase P_a_O_2_/F_i_O_2_ ratio [54, 55] by limiting the degree of bronchospasm. In addition, epinephrine decreases blood flow to injured/obstructed airways, thus improving V/Q matching.

Muscarinic receptor antagonists such as tiotropium have been studied as well. The parasympathetic response, mediated via muscarinic receptors in the lung, causes smooth muscle constriction within the airways, release of cytokines, and stimulation of submucosal glands [2]. Therefore, by inhibiting these effects, airway pressures are decreased and mucus secretion and cytokine expression are reduced [56]. Jonkam *et al.* demonstrated in an ovine model that tiotropium improved P_a_O_2_/F_i_O_2_ ratios and decreased peak airway pressures in the first 24 h following inhalation injury [56].

There is also evidence that both beta agonists and muscarinic receptor antagonists may decrease the host inflammatory response. Additionally, both muscarinic and adrenergic receptors are found on respiratory epithelial gland cells and may impact regeneration and healing following injury. Jacob *et al.* [57] showed in an ovine model that albuterol/tiotropium resulted in an increase in bronchial ciliated duct and submucosal gland cell proliferation following smoke inhalation and burn injury. In healthy human volunteer and animal studies, epinephrine has been shown to decrease tumor necrosis factor-alpha levels and potentiate IL-10 (a cytokine inhibitor) after lipopolysaccharide stimulation [58, 59].

### Mucolytic agents

N-acetylcysteine (NAC) is a powerful mucolytic and may have a role in mitigating ROS damage as it is a precursor of glutathione and a strong reducing agent. While it may aid in breaking up thick airway secretions, it is also an airway irritant and may produce bronchoconstriction; therefore, patients are frequently pre-dosed with a bronchodilating agent [38]. NAC has been proven to be effective in combination with aerosolized heparin for the treatment of inhalation injury in animal studies [60].

### Anticoagulants

Inhaled anticoagulants have been used to ameliorate the formation of fibrin casts, which contribute to airway obstruction following inhalation injury. This became a prevalent treatment after Desai *et al.* [61] demonstrated its utility in a pediatric inhalation injury population. However, in a subsequent retrospective review by Holt *et al.* [62], a cohort of 150 patients with inhalation injury showed no significant improvement in clinical outcomes in patients treated with inhaled heparin and acetylcysteine. This retrospective study allowed for the institution of nebulized heparin every 4 h for up to 7 days at the attending physician's discretion, and it is unclear whether significant selection bias impacted results [62]. There has been at least one case report of coagulopathy in a patient receiving nebulized heparin and acetylcysteine for inhalation injury [63]. However, Yip *et al.* [64] demonstrated that nebulized heparin does not increase the risk for pulmonary or systemic bleeding.

Miller *et al.* [65] found in a retrospective study that patients with inhalation injury who received nebulized heparin and NAC in addition to albuterol experienced a survival benefit with a number needed to treat of 2.73. A multi-center randomized controlled trial by Glas *et al.* [66] is currently underway to assess nebulized heparin versus placebo in inhalation injury.

Enkhbaatar *et al.* [67] used a combination of aerosolized heparin and recombinant human antithrombin in an ovine model of cutaneous burn and smoke inhalation. They found the two agents resulted in better lung compliance, less pulmonary edema, and less airway obstruction than controls. Interestingly, neither agent used alone had the same effect [67]. Using this same injury model, they demonstrated that a fibrinolytic agent, tissue plasminogen activator, decreased pulmonary edema, airway obstruction and airway pressures and improved gas exchange [68].

A systematic review of inhaled anticoagulants, including heparin, heparinoids, antithrombin, and fibrinolytics, in inhalation injury confirmed improved survival and decreased morbidity in preclinical and clinical studies [69]. Additionally, anticoagulants may have a systemic role in mitigating host inflammatory response. Combined burn and smoke inhalation injury is associated with myocardial impairment similar to septic cardiomyopathy. Rehberg *et al.* [70] demonstrated a decrease in the inflammatory changes underlying myocardial dysfunction and improvement in contractility in an ovine model following administration of recombinant human antithrombin.

### Anti-inflammatory agents

More specific therapies to mitigate the host inflammatory response and the positive feedback loop introduced through neutrophil migration into the airway and production of ROS and peroxynitrite (ONOO^−^) is an area of intense interest [71]. Neutralization of peroxynitrite with peroxynitrite decomposition catalysts has been demonstrated to be cytoprotective and provide beneficial effects in an ovine model of smoke inhalation injury. Hamahata *et al.* [72] demonstrated that peroxynitrite decomposition catalyst delivery into the bronchial artery of sheep subjected to burns and inhalation injury attenuated pulmonary damage when compared with a control group that received saline. Additionally, in an animal and human *in vitro* model of smoke inhalation injury, Perng *et al.* [25] demonstrated that NOS-mediated activation of the host inflammatory response was attenuated by inhibiting a specific signaling pathway (adenosine-monophosphate-activated protein kinase).

## Systemic toxicities

CO has an affinity for hemoglobin 200 to 250 times greater than oxygen and exposure results in hypoxia and ischemia. Unlike inhalation injury, CO has deleterious effects at the level of hemoglobin and more specifically the ability for oxygen delivery. CO acts to displace oxygen from hemoglobin (forming carboxyhemoglobin (COHb)) and binds to cytochrome c oxidase. COHb shifts the oxygen dissociation curve to the left, ultimately leading to decreased oxygen delivery at the tissue level and interfering with cellular respiration at the mitochondrial level [73]. Symptoms of CO toxicity include confusion, stupor, coma, seizures, and myocardial infarction [74]. CO diagnosis requires the use of a Co-oximeter (not available in every blood gas lab), since elevated COHb levels may be present despite normal P_a_O_2_ and oxygen saturation readings. Available since 2005, newer, non-invasive CO-oximetry monitors permit more rapid diagnosis [75]. CO poisoning is associated with an increased risk of mortality even at long-term follow-up (median of 7.6 years) [74]. Complications of CO poisoning include persistent and delayed neurologic sequelae and myocardial injury, as well as functional effects on leukocytes, platelets, and vascular endothelium [73]. Treatment of CO poisoning involves providing 100 % oxygen, which shortens the half-life of COHb to about 45 min.

Hyperbaric oxygen therapy (HBO) has been used to treat CO poisoning and can further reduce the COHb half-life to about 20 min. The benefits of HBO were evident in a study of 75 patients with acute CO poisoning. Three treatment sessions were administered within 24 h and neuropsychological tests were administered at various points throughout the study. They found the rates of cognitive sequelae were reduced at 6 weeks and 12 months after CO poisoning in patients with three HBO sessions [76]. It should be pointed out that the theoretical basis for HBO up to 24 h after exposure is to facilitate the clearance of CO from cytochrome c oxidase in the brain, rather than to increase its clearance from hemoglobin in the blood. Logistical factors have limited the utilization of HBO. A systematic review found that not enough evidence exists at this point to determine definitively whether HBO reduces adverse neurologic outcomes after CO poisoning [77]. Continued advancements in HBO technology combined with increased ICU accessibility will likely result in the generation of more clinical studies in this area.

The gaseous form of cyanide, hydrogen cyanide (HCN), is formed in fire atmospheres from the thermal decomposition of nitrogen-containing polymers, both natural (wool, silk, and paper), and synthetic (nylon and polyvinyl chloride). The significance of HCN in fire environments is unclear [11]. The lack of a rapid and reliable test to detect cyanide poisoning limits our understanding of the role of HCN in inhalation injury [78]. Additionally, symptoms can mimic CO poisoning. Dumestre *et al.* [79] found that most burn centers do not test for cyanide poisoning on admission and do not administer an antidote on the basis of clinical suspicion alone. Lactate has been suggested as a marker for severity of cyanide poisoning without other comorbidities [80], but its role in inhalation injury is less clear in a population at risk for CO poisoning and with coexisting hypovolemic shock. Hydroxocobalamin, the most commonly available antidote (sold as Cyanokit®), binds to HCN to form cyanocobalamin, which is nontoxic and excreted in the urine. The standard dose of 5 g is infused intravenously over 15 min. A second dose of 5 g can be administered in patients with severe toxicity or poor clinical response. It is generally regarded as safe. Red discoloration of the skin and urine is common, which may interfere with colorimetric assays.

Sodium nitrite (300 mg) and sodium thiosulfate (12.5 g) are also commercially available (sold as Nithiodote™). Prior to 2007, when hydroxocobalamin became available, sodium nitrite and sodium thiosulfate were used primarily for cyanide poisoning despite limited data as to their efficacy. In 2012, Bebarta *et al.* [81] evaluated sodium thiosulfate versus hydroxocobalamin in a swine model of severe cyanide poisoning. They found that sodium thiosulfate failed to reverse cyanide-induced cardiovascular collapse. Further, sodium thiosulfate was not found to be effective when added to hydroxocobalamin. Hydroxocobalamin alone was found to be effective for severe cyanide toxicity. Treatment with nitrites carries significant risk of hypotension and methemoglobinemia, which can further jeopardize tissue oxygen delivery.

Complications from inhalation injuryInhalation injury can be divided into anatomic levels and the mechanism of injury - direct thermal injury to the upper airways or chemical injury to the subglottic region and tracheobronchial tree [82–84]. The associated complications vary with the level of injury and are also effected by intubation, infection, and chronic inflammation [82]. In addition, the complications from injury may be acute or delayed. Pneumonia and airway obstruction are early complications of inhalation injury and have been well described in the literature [4, 8]. However, there is a paucity of data on the long-term or delayed complications from inhalation injury [84].

### Pneumonia

The most common complication following inhalation injury is respiratory tract infection [85]. Thermal injury activates the host inflammatory response which, when coupled with direct pulmonary injury, places the respiratory system at risk for infection. There is also evidence inhalation injury damages ciliated cells and causes them to detach from the airway epithelium [86]. Coupled with the exfoliation of airway epithelium by chemical irritation, the loss of ciliated cells impairs pulmonary immune function [85, 86]. Surfactant production is also impaired [87] as is mucociliary transport secondary to damage to airway epithelium [88]. The development of respiratory tract infection is also effected by decreased function of pulmonary macrophages [89]. Once the diagnosis of pneumonia is made, empiric antibiotics should be immediately administered. The antibiotic regimen should then be tailored based on the final sputum culture.

At this institution, 1,058 burn patients were evaluated with 35 % diagnosed with inhalation injury via bronchoscopy or ^133^Xenon lung scan [8]. Of these patients, 38 % developed pneumonia compared with 8.8 % in those without inhalation injury. These authors reported an estimated 20 % increase in mortality with burns and concomitant inhalation injury; mortality increased to 60 % with the development of pneumonia. They found inhalation injury and pneumonia to be independent risk factors for mortality [8].

### Airway obstruction

With direct injury to airway epithelium and fluid shifts, upper airway obstruction and pulmonary edema can occur [83]. Airway obstruction is further exacerbated by large fluid resuscitations and should be avoided [83]. Approximately one-third to one-fifth of patients with inhalation injury suffer from acute airway obstruction due to injury to supraglottic structures [38]. These patients require a secure airway either by intubation or tracheostomy [90]. Musosal edema usually peaks around 24 h post-burn and slowly improves over the following several days [90, 91].

From intubation, certain acute complications are known - barotrauma and suction-related injuries - which place the patient more at risk for hospital-acquired pneumonia [90]. Delayed consequences of intubation include tracheomalacia, subglottic stenosis, or innominate fistula [38, 43, 90, 92]. Complications associated with tracheostomies include bleeding, tube malposition, tracheal ulcerations, and tracheitis [91].

### Subglottic stenosis and other complications

Direct thermal injury below the vocal cords is unusual given the heat dissipation that occurs in the upper airways [93]. It is the particulate matter from the smoke inhalation and inhalation of steam that contributes significantly to the inflammatory cascade below the larynx [84, 91, 93, 94] and the formation of scar tissue or polyps.

Endobronchial polyps have been reported as both acute and delayed consequences of inhalation injury [84]. The etiology of polyps has been attributed to the epithelialization and fibrous replacement of granulation tissue after damage to the mucosal surfaces. Prevalence is unknown and development can be acute or delayed [84].

A retrospective review by Yang *et al.* [95] evaluated the incidence of tracheal stenosis in 1,878 burn patients. They found 0.36 % (seven patients) developed tracheal stenosis with five of them having FOB-confirmed inhalation injury (5.5 %). The average time to development was 7 months post-burn. Six patients required intubation for either respiratory distress or prophylaxis. They found prolonged intubation, the presence of inhalation injury, repeated intubates, and neck scar contractures impacted the development of tracheal stenosis [95]. Given the delayed development of stenosis, patients are at risk even after discharge and the true rate is unknown as patients can be symptom free. Other studies report a higher rate of tracheal stenosis in patients with inhalation injury (24 % [96] and 53 % [97]), and predominates in those who underwent intubation [98].

Other complications associated with inhalation injury are bronchiectasis [82, 99, 100], bronchiolitis obliterans [100], vocal cord fixation or fusion [82, 101], and dysphonia [102]. In order to identify and monitor the development of these complications, long-term follow-up (pulmonary function testing and FOB) is necessary [82, 98].

## Conclusion

Inhalation injury remains a significant cause of morbidity and mortality in thermally injured patients. Treatment of inhalation injury remains largely supportive. Recent research has led to substantial gains in the understanding of the molecular pathophysiology of inhalation injury. These advances as well as preclinical studies on targeted therapies provide hope for reversal of specific mechanisms of morbidity and improvement in outcomes.

## Abbreviations

APRV: Airway pressure release ventilation
ARDS: Acute respiratory distress syndrome
CO: Carbon monoxide
COHb: Carboxyhemoglobin
CT: Computed tomography
ECMO: Extracorporeal membrane oxygenation
F_i_O_2_: Fraction of inspired oxygen
FOB: Fiberoptic bronchoscopy
HBO: Hyperbaric oxygen therapy
HCN: Hydrogen cyanide
HFPV: High-frequency percussive ventilation
HTV: High tidal volume
IL: Interleukin
IL-1RA: IL-1 Receptor antagonist
LTV: Low tidal volume
NAC: N-acetylcysteine
NOS: Nitric oxide synthase
P_a_O_2_: Arterial partial pressure of oxygen
RADS: Radiologist’s score
ROS: Reactive oxygen species
